# Usefulness of Intracardiac Echocardiography-Guided Endomyocardial Biopsy for Diagnosing Transthyretin Cardiac Amyloidosis During Catheter Ablation

**DOI:** 10.1016/j.cjco.2025.05.011

**Published:** 2025-05-27

**Authors:** Maoko Atsumi, Koji Sudo, Kenji Kuroki, Akira Sato

**Affiliations:** Department of Cardiovascular Medicine, Faculty of Medicine, University of Yamanashi, Yamanashi, Japan

**Keywords:** atrial fibrillation, endomyocardial biopsy, intracardiac echocardiography, transthyretin cardiac amyloidosis

A 73-year-old female patient was referred to our hospital with worsening heart failure due to atrial fibrillation (AF) (Fig. 1A). Transthoracic echocardiography revealed a preserved left ventricular ejection fraction of 62%, with mild concentric myocardial hypertrophy (Fig. 1B; Video 1, view video online). Her history of carpal tunnel syndrome, heart failure with AF, myocardial hypertrophy, and an elevated troponin I level (28.5 pg/mL) raised concerns about the possibility of cardiac amyloidosis (CA). After 99mTc-pyrophosphate scintigraphy, transthyretin cardiac amyloidosis (ATTR-CA) was strongly suspected (Fig. 1C). We performed an endomyocardial biopsy (EMB) using a 3-dimensional (3D) delivery sheath (Selectra 3D, Biotronik, Berlin, Germany), as reported previously.^1^^,^^2^ The 3D delivery sheath and bioptome were inserted via the right jugular vein, and EMB was performed from the lower right ventricular septum (RVS), targeting the area where accumulation was observed on scintigraphy. Dual real-time imaging—using intracardiac echocardiography (ICE) at the right ventricular outflow, to visualize the bioptome located in the RVS (Fig. 1, D-F; Video 2, view video online), and angiographic images that included both right and left anterior oblique views (Fig. 1, G and H)—was used to safely perform EMB. Two tissue samples were obtained. After the EMB, transseptal puncture was performed using ICE, and pulmonary vein isolation was achieved using a pulsed-field ablation system (Farapulse, Boston Scientific, Marlborough, MA), as shown on the fluoroscopic image (Fig. 1I). A pathologic diagnosis using Congo-red and direct fast scarlet (DFS) staining revealed amyloidosis deposits and confirmed a diagnosis of ATTR-CA (Fig. 1J). The patient was treated with tafamidis meglumine as optimal medical therapy. EMB using a 3D delivery system under ICE guidance is a safe method for visualizing the RVS and reducing the risk of tricuspid valve leaflet avulsion. When performing catheter ablation for AF, we recommend performing an EMB if the patient has “red flags” for suspected CA.

**Novel Teaching Points**

• ATTR-CA might be overlooked in arrhythmia patients who are undergoing catheter ablation for AF. When a patient has “red flags,” such as myocardial hypertrophy or troponin-positive findings, a diagnosis of ATTR-CA should be considered.
• Myocardial hypertrophy and carpal tunnel syndrome should create suspicion of CA; 99mTc-pyrophosphate scintigraphy and EMB are recommended to diagnose and treat the CA.
• All the individual steps (endomyocardial biopsy, use of ICE, and catheter ablation) have been described previously and are part of the clinical routine, but they have not been combined these before. Thus, this case shows other clinicians what is possible.

**Figure 1:**
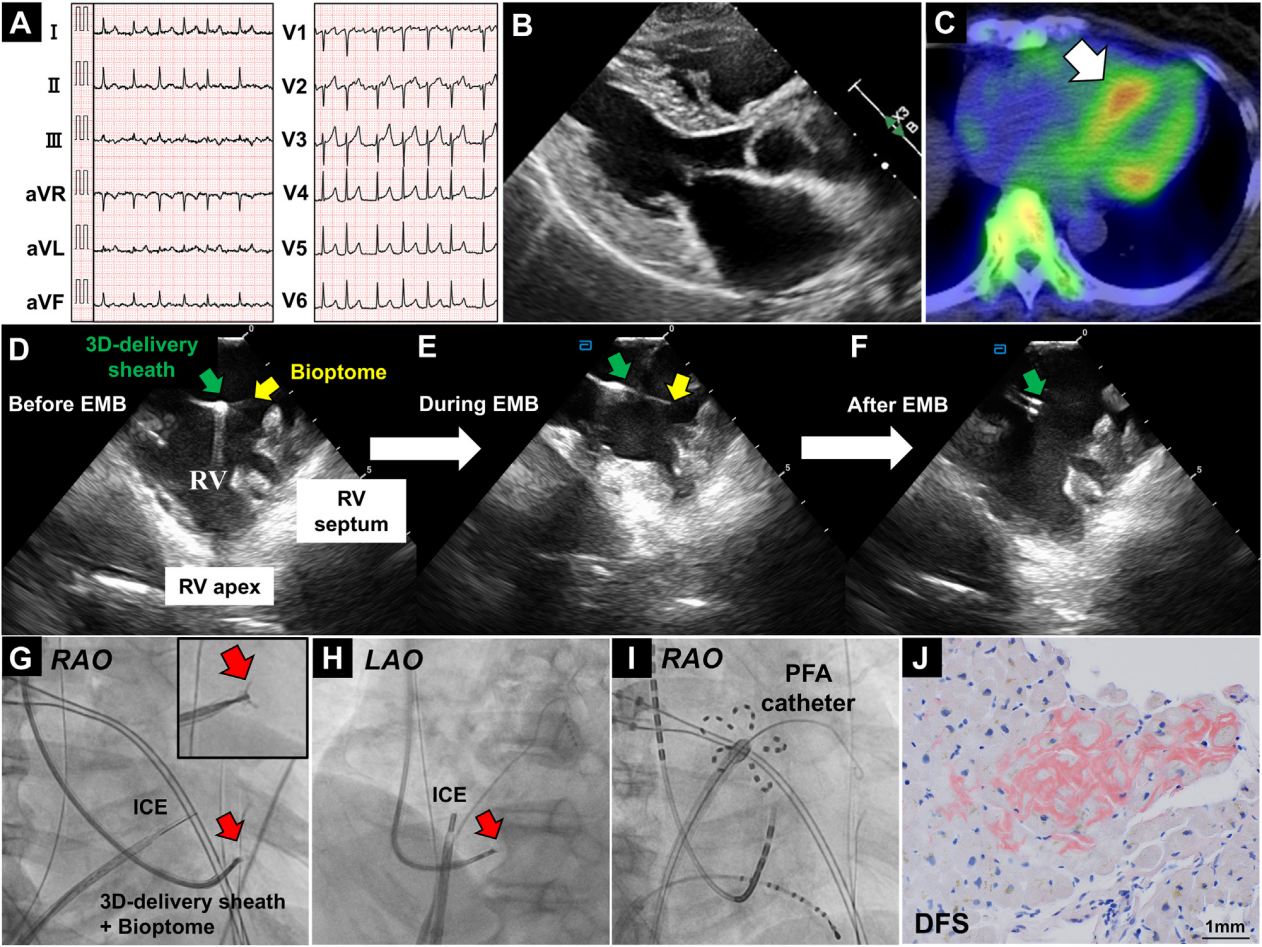
(**A**) Twelve-lead electrocardiography of atrial fibrillation. (**B**) Transthoracic echocardiography revealed a preserved left ventricular ejection fraction with mild concentric myocardial hypertrophy. (**C**) 99mTc-pyrophosphate scintigraphy showing accumulation at the mid-septal wall (**white arrow**). (**D-F**) Intracardiac echocardiography (ICE) at the right ventricular (RV) outflow to visualize the bioptome located in the RV septum. (**G, H**) Fluoroscopic images that included both the right anterior oblique (RAO) and left anterior oblique (LAO) views. (**I**) Fluoroscopic image during pulsed-field ablation (PFA). (**J**) Pathologic diagnosis using direct fast scarlet (DFS) staining revealing amyloidosis deposits. EMB, endomyocardial biopsy; 3D, 3-dimensional.

## Supplementary Material

Video 1 Transthoracic echocardiography revealed a preserved left ventricular (LV) ejection fraction of 62% with mild concentric myocardial hypertrophy.

Video 2 Real-time imaging was performed using intracardiac echocardiography (ICE) at the right ventricular outflow to visualize the bioptome located in the right ventricular septum.
